# MicroRNA-574-3p, identified by microRNA library-based functional screening, modulates tamoxifen response in breast cancer

**DOI:** 10.1038/srep07641

**Published:** 2015-01-06

**Authors:** T. Ujihira, K. Ikeda, T. Suzuki, R. Yamaga, W. Sato, K. Horie-Inoue, T. Shigekawa, A. Osaki, T. Saeki, K. Okamoto, S. Takeda, S. Inoue

**Affiliations:** 1 Division of Gene Regulation and Signal Transduction, Research Center for Genomic Medicine, Saitama Medical University, Saitama, Japan; 2 Department of Obstetrics and Gynecology, Juntendo University School of Medicine, Tokyo, Japan; 3 Departments of Pathology and Histotechnology, Tohoku University, Graduate School of Medicine, Sendai, Japan; 4 Departments of Geriatric Medicine, Graduate School of Medicine, The University of Tokyo, Tokyo, Japan; 5 Departments of Anti-Aging Medicine, Graduate School of Medicine, The University of Tokyo, Tokyo, Japan; 6 Department of Breast Oncology, International Medical Center, Saitama Medical University, Saitama, Japan; 7 Division of Cancer Differentiation, National Cancer Center Research Institute, Tokyo, Japan

## Abstract

Most primary breast cancers express estrogen receptor α and can be treated via endocrine therapy using anti-estrogens such as tamoxifen; however, acquired endocrine resistance is a critical issue. To identify tamoxifen response-related microRNAs (miRNAs) in breast cancer, MCF-7 cells infected with a lentiviral miRNA library were treated with 4-hydroxytamoxifen (OHT) or vehicle for 4 weeks, and the amounts of individual miRNA precursors that had integrated into the genome were evaluated by microarray. Compared to the vehicle-treated cells, 5 ‘dropout' miRNAs, which were downregulated in OHT-treated cells, and 6 ‘retained' miRNAs, which were upregulated in OHT-treated cells, were identified. Of the dropout miRNAs, we found that miR-574-3p expression was downregulated in clinical breast cancer tissues as compared with their paired adjacent tissues. In addition, anti-miR-574-3p reversed tamoxifen-mediated suppression of MCF-7 cell growth. Clathrin heavy chain (CLTC) was identified as a miR-574-3p target gene by in silico algorithms and luciferase reporter assay using the 3′ untranslated region of CLTC mRNA. Interestingly, loss and gain of miR-574-3p function in MCF-7 cells causes CLTC to be upregulated and downregulated, respectively. These results suggest that functional screening mediated by miRNA libraries can provide new insights into the genes essential for tamoxifen response in breast cancer.

Breast cancer is one of the most common malignancies leading to cancer-related mortality in women^1^. Approximately 70–80% of primary breast cancers express estrogen receptor α (ERα) and are considered to be regulated by estrogen^2^. Tamoxifen is an ERα antagonist that competitively inhibits the interaction of estrogen with ERα^3^ thus, most breast cancers can be treated with endocrine therapy using anti-estrogens such as tamoxifen or aromatase inhibitors after surgery or radiation for primary prevention strategy^4^,^5^. Nevertheless, ~40% of early-stage breast cancer patients who receive tamoxifen as an adjuvant therapy eventually relapse with tamoxifen-resistant disease^6^. Thus, acquired endocrine resistance is a critical issue for the management of breast cancer.

The molecular mechanisms underlying endocrine resistance in terms of its key regulators and signaling events remain to be elucidated. As one of the new transcriptional regulators involved in cancer biology, particular attention has been paid to the dysregulation of microRNAs (miRNAs) in tumor progression, including metastatic and angiogenic states^7^,^8^,^9^. miRNAs are small noncoding RNAs consisting of 20–22 nucleotides. They mostly bind to 3′-untranslated regions (3′-UTRs) of mRNAs at sequences that have imperfect or perfect complementarity, leading to posttranscriptional silencing of the targeted genes^10^.

Recent studies have suggested that miRNAs may contribute to the acquisition of tamoxifen resistance. Ward *et al.* reported that tamoxifen-resistant breast cancer cells derived from long-term passage of MCF-7 cells with tamoxifen exhibited a loss of miR-375 expression and had acquired epithelial-mesenchymal transition (EMT)-like properties. Whereas, re-expression of miR-375 sensitized the tamoxifen-resistant breast cancer cells to tamoxifen and partly reversed the EMT. In the report, *MTDH*, which encodes metadherin, was demonstrated to be a direct target of miR-375^11^. In another study, Bergamaschi *et al.* reported that miR-451 and its target 14-3-3ζ, a member of the 14-3-3 family, are associated with tamoxifen resistance^12^. Their study showed that tamoxifen upregulates 14-3-3ζ expression via the downregulation of miR-451. Overexpression of miR-451 could also recover the growth-inhibitory effect of tamoxifen on the proliferation of tamoxifen-resistant MCF-7 cells.

In the present study, we performed a miRNA library screen to identify miRNAs modulating tamoxifen responses in human breast cancer MCF-7 cells. By comparing miRNA expression in cells treated with 4-hydroxytamoxifen (OHT) to that in vehicle-treated cells, 5 ‘dropout' and 6 ‘retained' miRNAs were selected based on the fold change values of array signal intensities (by <0.2-fold for dropout miRNAs and >5-fold for retained miRNAs) and the coefficient of variation values (<60). One of the dropout miRNAs in the OHT-treated cells, miR-574-3p, was found to be downregulated in OHT-resistant MCF-7 cells (OHTR cells) as compared to parental MCF-7 cells, as well as in clinical breast cancer tissues compared to paired adjacent normal tissues. We then conducted growth assays on both parental cells and OHTR cells by transfecting them with either pre-miR-574-3p or anti-miR-574-3p. Knockdown of endogenous miR-574-3p revealed that this miRNA is critical for the tamoxifen response in MCF-7 cells. By *in silico* analysis for miRNA binding sites, *clathrin heavy chain* (*CLTC*) was identified as a candidate miR-574-3p target. Interestingly, loss and gain of miR-574-3p function in MCF-7 cells resulted in upregulation and downregulation, respectively, of *CLTC* mRNA. These results show that miRNA library-based functional screening can provide new insights into the genes essential for tamoxifen response in breast cancer and could be applied to the development of alternative options for breast cancer diagnosis and treatment.

## Results

### Screening for miRNAs affecting tamoxifen reactivity in breast cancer MCF-7 cells

**Figure 1:**
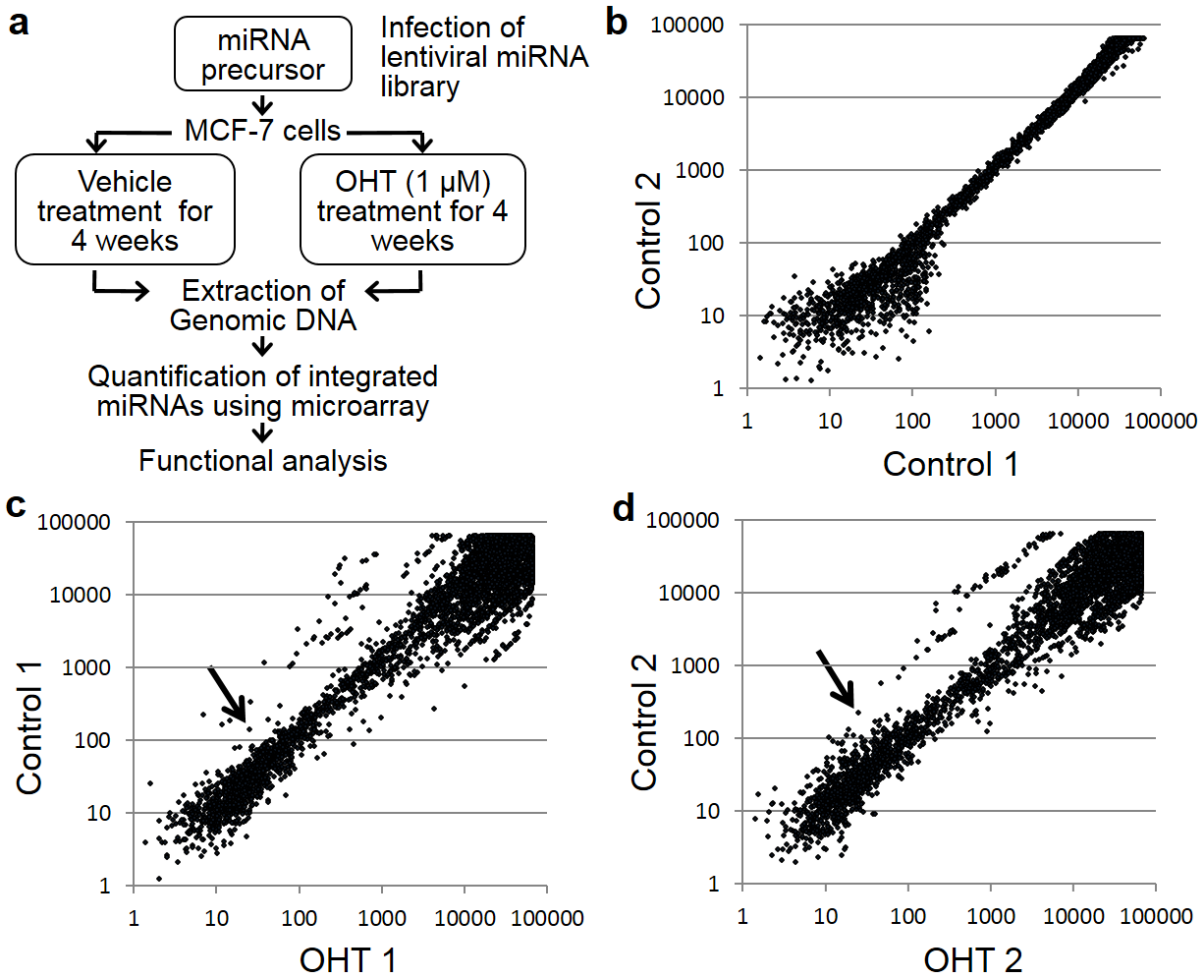
Screening of miRNAs associated with tamoxifen response in MCF-7 cells. (a) Schematic diagram of screen procedure using a lentiviral miRNA library to identify mediators of the tamoxifen response in human breast cancer MCF-7 cells. In brief, cells were infected with a lentiviral miRNA library and further cultured with or without the anti-estrogen 4-hydroxytamoxifen (OHT). Amounts of miRNAs integrated into genomic DNAs of surviving cells were quantified by Agilent's two-color CGH array platform. (b) Validation of miRNA screening reproducibility using 2 independent control experiments. Scatter plots of array signal intensities for individual miRNAs for Control 1 and 2 samples were generated using those signals for which the value of the coefficient of variation was less than 60. (c and d) Scatter plots of array signal intensities for individual miRNAs for OHT-treated and vehicle-treated samples (c, OHT sample 1 versus Control 1; d, OHT sample 2 versus Control 2). Arrows indicate the signals corresponding to miR-574-3p.

To identify miRNAs affecting tamoxifen response in MCF-7 cells, we utilized a lentiviral library consisting of 445 miRNA precursors. MCF-7 cells were infected with the library at different multiplicities of infection, and cell populations showing ~30% infection efficiency were selected and continuously cultured for 1 month in the presence of 1 μM OHT or vehicle (Figure 1a). We prepared 2 groups each of OHT-treated or vehicle-treated cells and then extracted genomic DNA from the surviving cells at the end of the cultivation period. The miRNAs that had integrated into the genome were amplified by PCR, using specific primers against the common sequences that flank each miRNA, and then quantified by microarrays using a two-color fluorescent probe hybridization system. The array signal plots comparing the 2 independent control samples were linearly distributed along a diagonal line (Figure 1b), indicating that the biological duplicates exhibited highly consistent results. In contrast, plots comparing the OHT-treated samples with the control samples were distributed in either the upper or lower areas flanking a diagonal line, showing that the miRNAs that had originally integrated into genomic DNA at the time of infection were either dropped out or retained, respectively, during the 1-month tamoxifen treatment period (Figure 1c and d). Using the following fold change values as criteria (<0.2-fold for dropout and >5-fold for retained), 5 dropout and 6 retained miRNAs were selected from our screening (Tables 1 and 2).

### miR-125a, miR-574-3p, and miR-877 are downregulated in both OHTR cells and clinical breast cancer tissues

**Figure 2:**
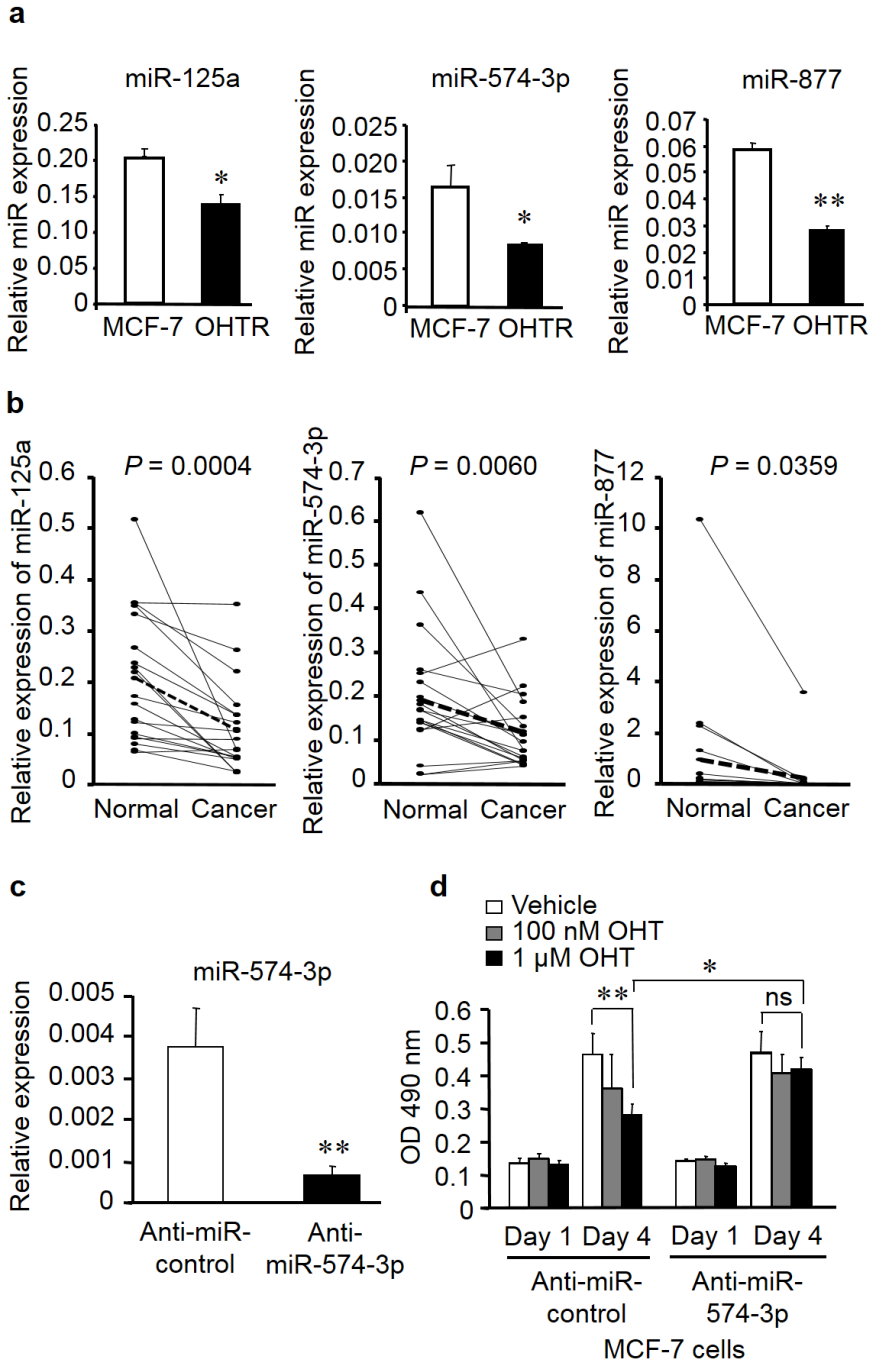
Downregulation of miR-125a, miR-574-3p, and miR-877 in 4-hydroxytamoxifen (OHT)-resistant MCF-7 cells (OTHR cells) and clinical breast cancer tissues, and knockdown of miR-574-3p–promoted MCF-7 cell growth in the presence of OHT. (a) Expression levels of miR-125a, miR-574-3p, and miR-877 in MCF-7 cells and OHTR cells were determined by quantitative PCR (qPCR) and normalized to *RNU48* levels. Data are presented as mean ± SE. *P < 0.05; **P < 0.01. (b) Decreased expression levels of miR-125a, miR-574-3p, and miR-877 in breast cancer tissues compared with those in paired adjacent normal tissues. (c) Knockdown efficiency of anti-miR-574-3p. MCF-7 cells were transfected with anti-miR-574-3p or negative control for 48 h. miR-574-3p levels were determined by qPCR and normalized to RNU48 levels. Data are presented as mean ± SE in triplicates. **P < 0.01. (d) Knockdown of miR-574-3p significantly increased MCF-7 cell growth in the presence of OHT. Cells were transfected with anti-miR-574-3p or negative control for 12 h, and then cell viability was analyzed using the MTS cell proliferation assay at 1 and 4 days after transfection. Data are presented as mean ± SE, in triplicate; *P < 0.05; **P < 0.01; ns, not significant.

Both overexpressed and underexpressed miRNAs could be involved in the transformation of tamoxifen sensitivity in MCF7 cells, putatively by repressing their distinctive target genes. In general, overexpressed miRNAs in cancers may function as oncogenes and promote cancer development by negatively regulating tumor suppressor genes whereas underexpressed miRNAs may function as tumor suppressor genes and inhibit cancers by regulating oncogenes^13^. In this context, we paid special attention to underexpressed tumor suppressive miRNAs that could regulate the expression of their oncogenic genes and have not been yet characterized. We found that one of the dropout miRNAs was let-7f, which is consistent with previous findings that let-7 family members function as tumor suppressors in breast cancer^14^,^15^,^16^,^17^. To determine endogenous expression levels of the dropout miRNAs, we generated OHTR cells by long-term culture with OHT. Quantitative PCR (qPCR) using RNAs prepared from MCF-7 and OHTR cells showed that expression levels of miR-125a, miR-574-3p, and miR-877 were significantly downregulated in OHTR cells as compared to parental cells (Figure 2a). miR-105-2 was excluded from the further experiments because it was not found to be significantly downregulated in OHTR cells by qPCR (data not shown). We next examined the expression levels of miR-125a, miR-574-3p, and miR-877 in clinical breast cancer samples composed of 19 paired samples of breast carcinoma and adjacent normal tissues from 19 patients (Figure 2b). As shown in the figure, levels of miR-125a, miR-574-3p, and miR-877 expression were significantly lower in breast cancer tissues than adjacent normal tissues. The results indicate that the downregulation of miR-125a, miR-574-3p, and miR-877 may correlate with the development and progression of tamoxifen resistance in breast cancer. Our results support the results from previous studies indicating that miR-125a plays a tumor suppressive role in human breast cancer^18^,^19^. We were particularly interested in miR-574-3p, as its *P* value was much lower than that of miR-877 in comparison between carcinoma tissues and adjacent normal tissues (Figure 2b). The role of miR-574-3p has not been elucidated in breast cancer though it has been reported to be a tumor suppressive miRNA in prostate cancer^20^. Nevertheless, the manner of miRNA selection used in the present study does not exclude the pathological relevance of other miRNAs that were not studied further in this work.

### Knockdown of miR-574-3p reverses tamoxifen-dependent suppression of cell growth in MCF-7 cells

To investigate the functional role of miR-574-3p in the proliferation of breast cancer cells, we performed loss-of-function studies using the miRNA inhibitor anti-miR-574-3p. The expression of miR-574-3p was significantly reduced in MCF-7 cells transfected with anti-miR-574-3p (Figure 2c). A cell viability assay showed that OHT treatment significantly repressed the growth of MCF-7 cells transfected with a control miRNA inhibitor. However, transfection of anti-miR-574-3p abrogated the OHT-mediated suppression of cell growth in MCF-7 cells (Figure 2d). We confirmed that the growth of MCF-7 cells was inhibited by OHT in a concentration-dependent manner, whereas that of OHTR cells was not repressed (Supplementary Figure S1). These results indicate that the downregulation of miR-574-3p contributes to tamoxifen resistance in breast cancer cells.

### Identification of candidate target genes for miR-574-3p

**Figure 3:**
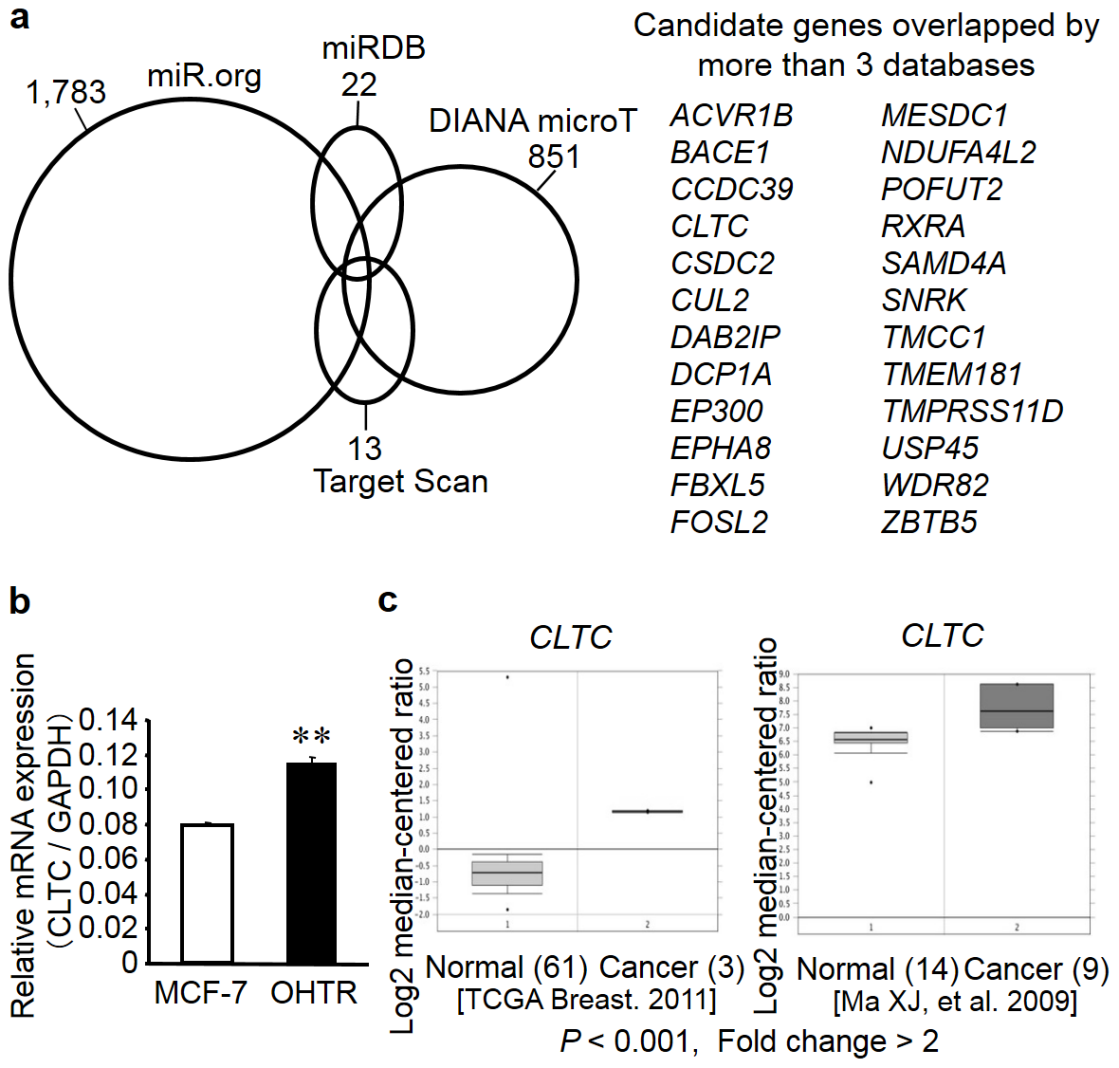
Identification of miR-574-3p target genes in breast cancer. (a) Schematic presentation of miR-574-3p target prediction by *in silico* analyses. Venn diagrams indicating numbers of candidate hits determined by 4 online prediction algorithms. Candidate genes commonly predicted by >3 algorithms are described. (b) *CLTC* mRNA was upregulated in 4-hydroxytamoxifen (OHT)-resistant MCF-7 cells (OHTR cells). Expression levels of *CLTC* mRNA were determined by quantitative reverse transcription PCR (qRT-PCR) in parental MCF-7 cells and OHTR cells. Data are presented as mean ± SE in triplicate; **P < 0.01. (c) *CLTC* mRNA was overexpressed in clinical breast cancer tissues compared to normal mammary tissues, based on the datasets in Oncomine cancer profiling database (reported by The Cancer Genome Atlas Breast 2011 and Ma *et al*.^44^ by >2-fold at *P* < 0.001).

To identify candidate targets of miR-574-3p, we used 4 target gene prediction programs: TargetScan^21^, DIANA-microT^22^, miRDB^23^, and miR.org^24^ (Figure 3a). TargetScan identified 1,783 candidates, DIANA-microT identified 851 candidates, miRDB identified 22 candidates, and miR.org identified 13 candidate targets for miR-574-3p. Notably, 24 genes (*ACVR1B, BACE1, CCDC39, CLTC, CSDC2, CUL2*, *DAB2IP, DCP1A, EP300, EPHA8, FBXL5, FOSL2, MESDC1, NDUFA4L2, POFUT2,* *RXRA,* *SAMD4A, SNRK, TMCC1, TMEM181, TMPRSS11D, USP45, WDR82,* and *ZBTB5)* were present in at least 3 of the 4 databases. Of these candidates, we focused on *CLTC* because it has been shown to be involved in tumorigenesis of hepatocellular carcinoma and pancreatic cancer^25^,^26^. Indeed, the level of *CLTC* mRNA expression was higher in OHTR cells than in the parental MCF-7 cells (Figure 3b). We then examined the Oncomine microarray database to determine whether *CLTC* expression was altered in clinical breast cancer samples^27^. Two datasets indicated that *CLTC* was substantially overexpressed in clinical breast cancers compared to normal breast tissues (by >2-fold; *P* < 0.001; Figure 3c).

### miR-574-3p regulates *CLTC* mRNA expression

**Figure 4:**
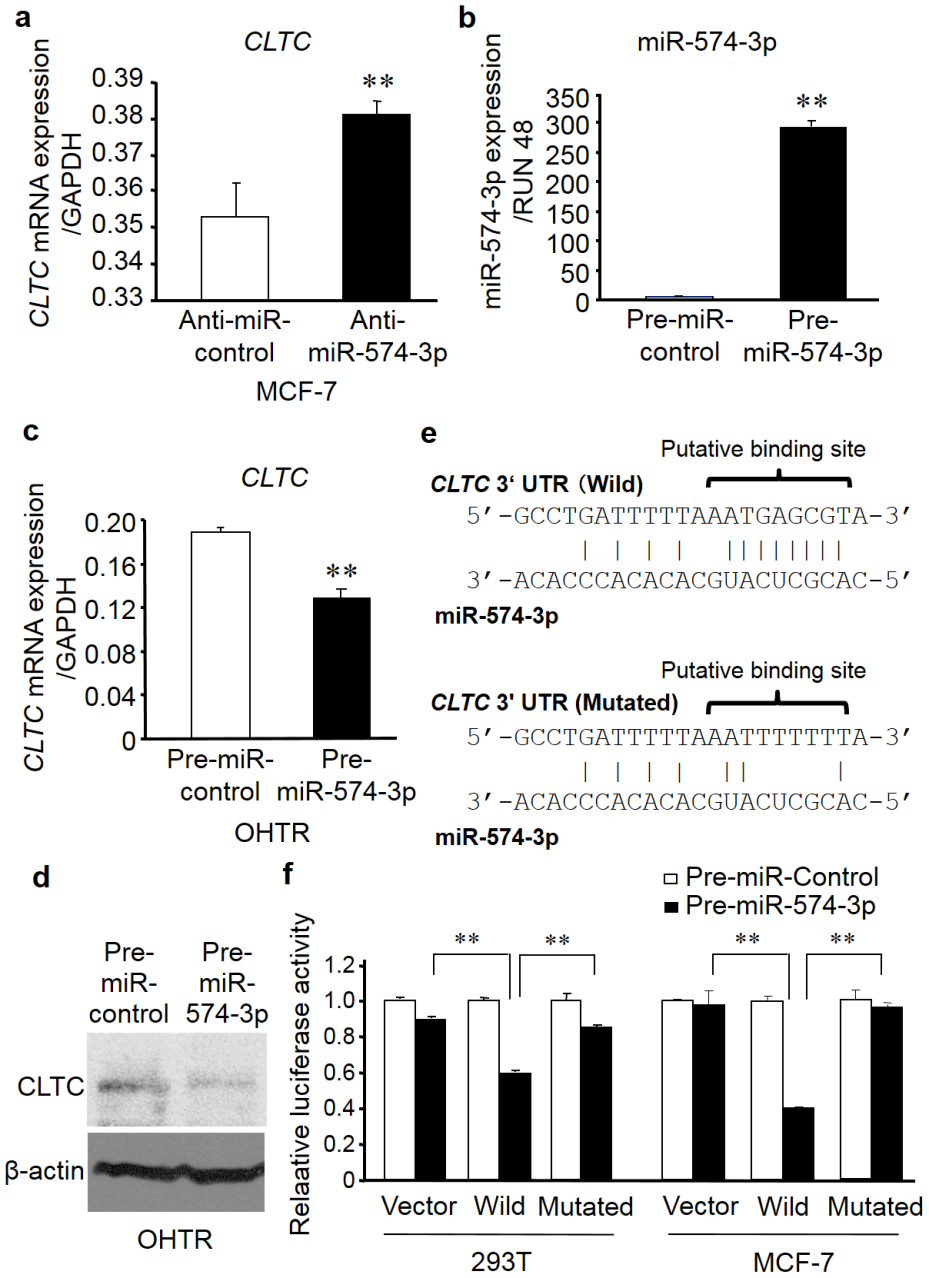
miR-574-3p modulates its candidate target *CLTC* mRNA expression in MCF-7 cells. (a) Silencing of miR-574-3p induces *CLTC* mRNA expression. MCF-7 cells were transfected with anti-miR-574-3p or control anti-miR for 48 h, and then expression levels of miR-574-3p were evaluated by qPCR. Data are presented as mean ± SE; **P < 0.01. (b) Validation of miR-574-3p overexpression by transfection of the miR-574-3p precursor. Cells were transfected with pre-miR-574-3p or control pre-miR for 48 h, and then the expression levels of miR-574-3p were evaluated by qPCR. Data are presented as mean ± SE; **P < 0.01. (c) Overexpression of miR-574-3p decreased *CLTC* mRNA expression. Cells were transfected with pre-miR-574-3p or control pre-miR for 48 h, and then the expression levels of miR-574-3p were evaluated by qPCR. Data are presented as mean ± SE; **P < 0.01. (d) Overexpression of miR-574-3p decreased CLTC protein expression. Cells were transfected with pre-miR-574-3p or control pre-miR for 48 h, and then the expression levels of CLTC protein were evaluated by western blot using anti-CLTC antibody. Loading control was obtained with anti-β-actin antibody. (e) Location of putative miR-574-3p-binding sequences and mutated sites in the 3′-UTR of target genes. (f) Luciferase reporter assay using vectors containing a putative *CLTC* 3′-UTR binding site for miR-574-3p and a mutated version of the site. The 293T and MCF-7 cells were transiently transfected with psiCHECK2 vectors containing either the wild-type or mutated putative binding sites for miR-574-3p, together with pre-miR-574-3p precursor or control pre-miR for 48 h, and then the luciferase assay was performed. Data are presented as mean ± SE; **P < 0.01.

To determine whether miR-574-3p directly regulates *CLTC* expression, we transfected MCF-7 cells with anti-miR-574-3p for 48 h and then evaluated the level of *CLTC* mRNA by quantitative reverse transcription polymerase chain reaction (qRT-PCR). Transfection with anti-miR-574-3p increased the level of *CLTC* mRNA expression by 2-fold relative to the control anti-miR (Figure 4a). Next, we transfected OHTR cells with pre-miR-574-3p for 48 h and then quantified the level of *CLTC* mRNA and protein. Transfection with pre-miR-574-3p increased the level of miR-574-3p expression (Figure 4b) and resulted in a significant reduction in the level of *CLTC* mRNA and protein levels in OHTR cells, as compared to OHTR cells transfected with a control miRNA precursor (Figure 4c and d).

### Putative miR-574-3p binding site in the *CLTC* 3′-UTR

We then computationally analyzed the 3′-UTR of *CLTC* to identify potential recognition sites for miR-574-3p. One of the target prediction database online systems, miRDB (http://mirdb.org/miRDB/)^23^, predicted a single recognition sequence containing a conserved 7-mer exact seed match at positions 573–579 bp (Figure 4e) in the *CLTC* 3′-UTR. This result indicates that miR-574-3p may directly bind to *CLTC* 3′-UTR to regulate *CLTC* expression at the transcriptional level.

### *CLTC* is a target of miR-574-3p

Next, we constructed luciferase reporter vectors containing either the wild-type *CLTC* 3′-UTR sequence with the putative binding site for miR-574-3p or a version of the 3′-UTR in which the putative site had been mutated (Figure 4f). In both 293T and MCF-7 cells, the luciferase assay demonstrated that miR-574-3p decreased the activities of luciferase reporter with the wild-type sequence but not the mutated sequence. This result suggests that miR-574-3p regulates *CLTC* expression by binding to the 3′ UTR of *CLTC* mRNA (Figure 4f).

### *CLTC* siRNA knockdown restores tamoxifen sensitivity, and low *CLTC* levels are correlated with better survival in tamoxifen-treated breast cancer patients

**Figure 5:**
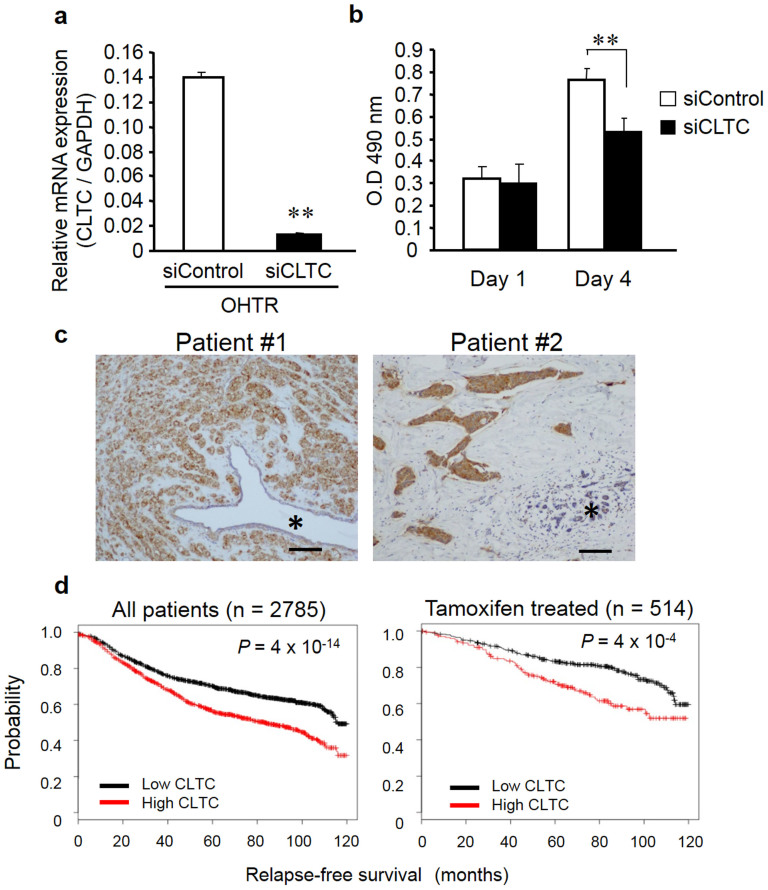
*CLTC* knockdown restored tamoxifen sensitivity, and low *CLTC* levels are correlated with better survival in breast cancer patients. (a) Knockdown efficiency of CLTC siRNA. OHTR cells were transfected with siCLTC or negative control for 48 h. *CLTC* mRNA levels were determined by qPCR and normalized to *GAPDH* levels. Data are presented as mean ± SE, in triplicate; **P < 0.01. (b) Knockdown of *CLTC* significantly reduced OHTR cell growth in the presence of OHT. Cells were transfected with siCLTC or negative control, and then cell viability was analyzed by MTS cell proliferation assay at 1 and 4 days after transfection. Data are presented as mean ± SE, in triplicate; **P < 0.01. (c) Representative immunohistochemical staining of breast cancer tissues with anti-CLTC antibody. CLTC immunoreactivity was predominantly detected in the cytoplasm of breast carcinoma cells compared with the epithelial cells of non-neoplastic glands. *; non-neoplastic mammary epithelium. Scale bar, 100 μm. (d) Kaplan–Meier curves of relapse-free survival times of total breast cancers (n = 2785), and patients with following endocrine therapy only using tamoxifen (n = 514), stratified by *CLTC* expression levels. This data were obtained from the Kaplan–Meier Plotter^25^.

To examine the functional role of *CLTC* in the tamoxifen response*,* we performed a loss-of-function study in OHTR cells using *CLTC* siRNA. We found that the level of *CLTC* mRNA expression was markedly repressed in OHTR cells transfected with *CLTC* siRNA as compared to the control (Figure 5a). In addition, a cell proliferation assay showed significant inhibition of proliferation in cells transfected with *CLTC* siRNA as compared to the control transfectants (Figure 5b). We also investigated whether *CLTC* siRNA effects on the growth of MCF-7 cells in the basal condition without tamoxifen. As a result, *CLTC* knockdown did not exhibit a substantial effect on the growth of the MCF-7 cells in the basal condition without tamoxifen (Supplementary Figure S2). Based on the data, we assume that CLTC will be more involved in tamoxifen resistance in breast cancer cells rather than in estrogen sensitivity in naïve breast cancer cells. We generated a CLTC expression plasmid and confirmed ectopic expression of CLTC protein in OHTR cells transfected with this plasmid. WST-8 assay showed that CLTC overexpression could increase the cell growth even in the presence of OHT, suggesting that CLTC may be associated with increasing the threshold of tamoxifen sensitivity. (Supplementary Figure S3). Furthermore, we performed immunohistochemical analysis of 10 breast cancer tissues using a CLTC-specific antibody (Figure 5c). CLTC immunoreactivity was predominantly detected in the cytoplasm of breast carcinoma cells, and it was also weakly observed in the epithelial cells of non-neoplastic glands adjacent to the carcinoma. When the positivity of CLTC was defined as stronger CLTC immunoreactivity in carcinoma cells versus their adjacent non-neoplastic epithelial cells, CLTC status was positive in 7 of 10 ER-positive breast carcinomas that we examined in the additional analysis (70%). The CLTC immunoreactivity was significantly higher in carcinomas compared to the non-neoplastic mammary glands (*P* = 0.018 by a Wilcoxon signed rank test). Interestingly, when we examined a breast cancer microarray dataset^28^, we found that low *CLTC* expression correlated with good prognosis, both in all patients examined and in those treated with tamoxifen monotherapy (Figure 5d). These results suggest that *CLTC* may play a role in malignant alteration, including the acquisition of tamoxifen resistance, in breast cancer.

## Discussion

In the present study, we performed a functional screen using a lentiviral miRNA library to identify miRNAs associated with acquired resistance for endocrine therapy in breast cancer. MCF-7 cells infected with the miRNA library were treated with tamoxifen or vehicle for one month, and then the profiles of the genome-integrated miRNAs from the 2 groups of cells were compared. Microarray analysis identified 5 dropout and 6 retained miRNAs in the OHT-treated cells. These miRNAs may be involved in the modulation of tamoxifen responses in MCF-7 cells.

In this study, we focused on the dropout miRNAs. miR-105 has been reported as a potential tumor suppressor in prostate cancer^29^, and miR-105 expression is decreased in prostate cancer cell lines as compared to normal prostate epithelial cells. In addition, miR-105 overexpression inhibited the growth of prostate cancer cells both *in vitro* and *in vivo*. Moreover, CDK6 has been identified as a putative target for miR-105. miR-877 has been shown to be one of the miRNAs induced by paclitaxel in hepatocellular carcinoma cells^30^.

miR-125a has been reported to be a potential tumor suppressor in breast cancer. miR-125a targets both *HER2* and *HER3*, and miR-125a overexpression in *HER2*-positive breast cancer cells leads to reductions in anchorage-dependent growth, cell motility, and invasiveness^18^,^19^. let-7f is a member of the let-7 family, whose expression is suppressed in various cancers^31^, and its restoration to normal levels has been reported to suppress cancer growth^32^,^33^. let-7f has also been demonstrated to directly target the aromatase gene and suppress cell proliferation and migration in breast cancer cells^34^. miR-574-3p has also been reported to be a tumor suppressor in prostate cancer^20^, bladder cancer^35^, and gastric cancer^36^.

We showed that miR-574-3p, miR-125a, and miR-877 were downregulated in clinical breast cancer tissues, suggesting that these miRNAs may play a role in breast cancer. Moreover, knockdown of endogenous miR-574-3p abrogated the tamoxifen-mediated growth suppression of MCF-7 cells. Therefore, we suggest that miR-574-3p modulates tamoxifen resistance in MCF-7 cells.

*CLTC* was identified as a potential target for miR-574-3p by an *in silico* screen of target genes. This computational finding is consistent with our results that *CLTC* mRNA levels were up- and downregulated in MCF-7 cells transfected with miR-574-3p inhibitor and precursor, respectively. A luciferase reporter assay demonstrated that miR-574-3p is able to decrease levels of *CLTC* mRNA expression. In addition, knockdown of *CLTC* using siRNA restored tamoxifen sensitivity in OHTR cells, and an examination of public microarray datasets revealed that low levels of *CLTC* expression correlated with better rates of survival for breast cancer patients.

*CLTC* encodes clathrin heavy chain, which, together with clathrin light chain, makes up clathrin. Clathrin, which occurs in a triskelion shape, is a major protein component of the cytoplasmic face of intracellular organelles^37^ and regulates endocytosis, via the clathrin-mediated endocytic pathway, and protein sorting^38^,^39^. *CLTC* thus plays a role in the uptake of ligand–receptor complexes, membrane transporters, and adhesion molecules^39^. It has also recently been reported that clathrin heavy chain localizes on the mitotic spindle and has an important role during mitosis^40^. A link between *CLTC* and tumorigenesis has been reported, and *CLTC* has been proposed as as a potential early detectable biomarker in hepatocellular carcinoma tissues^25^,^26^.

Clathrin heavy chain has been shown to promote tumor growth and hypoxia-induced angiogenesis by stabilizing hypoxia-inducible factor 1α and increasing vascular endothelial growth factor signaling^41^. In addition, Joffre *et al.* reported that clathrin knockdown decreased tumor growth and metastasis by inhibiting oncogenic Met, providing additional evidence that clathrin-mediated endocytic pathway may contribute to tumorigenesis^42^. As in the present study we found that mRNA levels of *CLTC* were upregulated in OHTR cells and breast cancer tissues. In addition, the effects of loss- and gain-of-function of *CLTC* were apparent in the presence of tamoxifen in OHTR cells. Thus, we assume that *CLTC* specifically modulates tamoxifen response in breast cancer cells via similar mechanisms.

In summary, we have identified miR-574-3p as a modulating factor for the tamoxifen response in breast cancer, based on miRNA library-based functional screening. A combination of *in silico* and *in vitro* analyses indicate that *CLTC* is a potential target of miR-574-3p. These findings can be used for the development of alternative options for breast cancer diagnosis and treatment.

## Methods

### Screening of lentiviral miRNA library and microarray analysis

Experimental concepts of our screen method were based on previous literature^43^. Briefly, a human miRNA precursor lentivirus library that coexpresses GFP was purchased from System Biosciences (Mountain View, CA, USA). This library contains a pool of 445 human miRNA precursor clones. MCF-7 cells were infected with the library at different multiplicities of infection together with 5 μg/mL polybrene. Transduction efficiency was evaluated by GFP expression 48 h after infection using FACS Calibur (Becton Dickinson, CA, USA).

To avoid the possibility of multiple infection, we selected cells with 29.3% (sample 1) and 30.9% (sample2) infection rates. Cells were continuously cultured in Dulbecco's modified Eagle's medium (DMEM) containing 1 μM 4 OHT or vehicle control (0.1% ethanol). During the culture period, medium was replenished every 2 to 3 days. After 4 weeks of culture, miRNA precursors integrated into the surviving cell populations were amplified by PCR using specific primers against the sequences in the lentivirus vector (forward primer: 5′-GCCTGGAGACGCCATCCACGCTG-3′; reverse primer: 5′-GATGTGCGCTCTGCCCACTGAC-3′), in order to amplify miRNA precursor sequences. PCR products from vehicle-treated and tamoxifen-treated MCF-7 cells were labeled with cyanine-3 or cyanine-5, respectively, using the Genome DNA Enzymatic Labeling Kit (Agilent) and then subjected to microarray hybridization (Oligo cDGH/ChIP-on-ChIP Hybridization Kit, Agilent). Agilent Feature Extractor software was used to scan microarray images and to normalize signal intensities.

Before comparing the microarray results between OHT-treated and control samples, the reproducibility of signal intensities was evaluated based on the calculation of the coefficient of variation (CV = 100 × SD/mean) in the duplicated OHT-treated and control experiments. Almost half of the signals had a CV less than 60 and were used for plotting. Signals showing <0.2-fold and >5-fold changes in the OHT-treated MCF-7 cells as compared to vehicle–treated cells were selected as candidate miRNAs potentially involved in the tamoxifen response.

### Cell culture and transfection of miRNA precursors and inhibitors

MCF-7, T47D, and 293T cells were purchased from ATCC (Manassas, VA, USA) and cultured in DMEM supplemented with 10% fetal bovine serum, 50 units/mL penicillin, and 50 μg/mL streptomycin at 37°C in a humidified atmosphere of 5% CO_2_. OHTR cells were established from MCF-7 cells by long-term (>3 months) culture with 1 μM OHT, and one of the clones exhibiting OHT-resistant proliferation was utilized for experiments. Pre-miR-574-3p and its positive control, as well as anti-miR-574-3p and its negative control, were purchased from Ambion (Carlsbad, CA, USA). Transfection of miRNA precursors or inhibitors was carried out using Lipofectamine RNAiMAX transfection reagent (Invitrogen, Carlsbad, CA, USA) according to the manufacturer's protocol.

### miRNA target prediction

Candidate targets for miR-574-3p were identified using 4 online database algorithms for miRNA target prediction: TargetScan (http://www.targetscan.org/)^21^, DIANA-microT (http://diana.cslab.ece.ntua.gr/microT/)^22^, miRDB (http://mirdb.org/miRDB/)^23^, and miRorg (http://www.microrna.org/microrna/getGeneForm.do)^24^.

### qPCR and qRT-PCR

Total RNA was extracted from cells using the ISOGEN reagent (Nippon Gene, Toyama, Japan). miRNA levels were determined by qPCR using triplicate Taqman microRNA assays (Applied Biosystems, CA, USA). The target gene mRNA levels were evaluated by the Step One Real-time PCR System (Applied Biosystems) using cDNAs converted from total RNA with SuperScript III Reverse Transcriptase (Invitrogen, Carlsbad, CA, USA). Results from 3 independent experiments were normalized to expression of endogenous *RNU48* for miRNA or *GAPDH* for mRNA, respectively. Primers for *CLTC* and *GAPDH* were as follows: CLTC forward: 5′- GAGCTTGTTGCTGAGGTTGAAA-3′, CLTC reverse: 5′-AGGCTCCTCACAGCCCTCAT-3′, GAPDH forward: 5′-GGTGGTCTCCTCTGACTTCAACA-3′, and GAPDH reverse: 5′-GTGGTCGTTGAGGGCAATG-3′.

### Cell growth assay

The effects of drugs or miRNAs on cell viability were determined by the MTS or WST-8 assay using the CellTiter 96 AQueous One Solution Cell Proliferation Assay kit (Promega, WI, USA) or Cell Count Reagent SF (NACALAI TESQUE, Kyoto, Japan). MCF-7 cells were cultured in 96-well plates at a density of 2,000 cells per well, and 10 μL of MTS solution was added to each well at the indicated time points (24, 72, or 96 h) after transfection. Cells were further incubated for 2 h at 37°C in a 5% CO_2_ incubator. The absorbance was measured at 490 nm with Multiscan FC Microplate Photometer (Thermo Fisher Scientific, MA, USA).

### Luciferase reporter assay

Oligonucleotides containing a putative binding site for miR-574-3p in the *CLTC* 3′-UTR (5′-TCGAGAGACAACTTGCCTGATTTTTAAA*TGAGCGT*AAAAGGCCCTGC-3′ and 5′-GGCCGCAGGGCCTTTT*ACGCTCA*TTTAAAAATCAGGCAAGTTGTCTC-3′) and its mutated sequences (5′-TCGAGAGACAACTTGCCTGATTTTTAAA**TTTTTTT** AAAAGGCCCTGC-3′ and 5′-GGCCGCAGGGCCTTTT**AAAAAAA** TTTAAAAATCAGGCAAGTTGTCTC-3′) were annealed, digested with *Eco*RI and *Xho*I, and cloned into the psiCHECK-2 vector (Promega). The sequences corresponding to putative miR-574-3p-binding site were shown in italic and mutated sequences were shown in bold. For the luciferase assay, 293T and MCF-7 cells were transfected with psiCHECK2 vector containing the wild-type or mutated putative binding sites for miR-574-3p, together with pre-miR-574-3p precursor or control pre-miR, using Lipofectamine 2000 transfection reagent (Invitrogen, CA, USA). The psiCHECK2 empty vector was also transfected as a mock control. The luciferase reporter assay was performed using the Dual-Luciferase Reporter Assay System (Promega) 48 h after transfection. The values for *Renilla* luciferase activity were normalized with the corresponding values for firefly luciferase activity. The experiments were performed in triplicate, and the results were expressed as mean ± SE.

### Clinical specimens

All clinical breast cancer tissues and the paired adjacent normal tissues were resected from patients at Saitama Medical University. We used 19 breast cancer specimens including 14 ER-positive samples and 5 ER-negative ones. Of 14 ER-positive breast cancers, 6 samples were collected from the patients treated with adjuvant tamoxifen. All procedures were performed under a protocol approved by the Ethics Committee at Saitama Medical University, and written informed consent was obtained from all patients. The methods were carried out in accordance with the approved guidelines.

Total RNA was isolated from these dissected samples and subjected to qRT-PCR analysis. Oncomine^TM^ Research Edition^27^ was used for the evaluation of *CTCL* mRNA expression in clinical breast cancer and normal mammary tissues based on microarray datasets. Kaplan–Meier curves of relapse-free survival times were obtained using the Kaplan–Meier Plotter (http://kmplot.com/analysis/), which is an online tool for the genome-wide validation of survival-associated biomarkers in breast, ovarian, and lung cancers using microarray data.

### Immunohistochemistry

For immunohistochemistry, 10 clinical breast cancer tissues were resected from patients at Saitama Medical University. Immunohistochemical analysis of CLTC expression was performed using an EnVision+ visualization kit (Dako, Carpinteria, CA). The tissue sections were deparaffinized, rehydrated through a graded ethanol series, and rinsed in Tris-buffered saline containing 0.05% Tween-20 (TBST). For antigen retrieval, the sections were heated at 100°C for 1 h in a 10 mM sodium citrate buffer (pH 6.0). The sections were blocked with endogenous peroxidase (0.3% H_2_O_2_) and incubated in 10% fetal bovine serum for 30 min. The anti-CLTC antibody (BD Biosciences, San Jose, CA) (1:500 dilution) was applied, and the samples were incubated overnight at 4°C. The sections were rinsed in TBST and incubated with EnVision+ horseradish peroxidase-labeled polymer for 1 h at room temperature. The antigen-antibody complex was visualized using a 3,3′-diaminobenzidine substrate kit for peroxidase (Vector Laboratories, Burlingame, CA).

### Expression plasmid for CLTC

The cDNA fragment containing the CLTC open reading frame (ORF) was amplified from cDNA synthesized from MCF-7 cells by PCR using the primers 5′-TTTGCGGCCGCTGGCCCAGATTCTGCCAATTCGTTTT-3′ and 5′- ACTGCGGCCGCTCACATGCTGTACCCAAAGCCAGG-3′. The CLTC ORF cDNA was C-terminally tagged with the Flag epitope and subcloned into the pcDNA3 vector (Promega). Western blot analysis was performed using the anti-CLTC and anti-β-actin (Sigma-Aldrich, St. Louis, MO) antibodies.

## Author Contributions

Conceived and designed the experiments: K.I. and S.I. Performed the experiments: T.U., K.I., T.S., W.S. and R.Y. Analyzed the data: K.H.-I., K.O. and S.T. Contributed reagents/materials/analysis tools: T.S., A.O., T.S. and K.O. Wrote the paper: T.U., K.I., K.H.-I. and S.I. All authors reviewed the manuscript.

## Supplementary Material

Supplementary Information Supplementary Figures

## Figures and Tables

**Table 1:** Dropout miRNAs after tamoxifen treatment

miRNA	Controla)	OHTb)	OHT/Control
miR-105-2	18526.7 ± 10013.5	459.3 ± 211.2	0.02
miR-877	5736.8 ± 1938.1	195.0 ± 29.9	0.03
let-7f	941.8 ± 351.1	57.3 ± 27.1	0.06
miR-125a	53507.6 ± 16213.9	3788.5 ± 381.2	0.07
miR-574-3p	136.9 ± 6.3	22.0 ± 4.3	0.16

^a)^Averaged signal intensity of miRNA in the vehicle-treated control cells was quantified by microarray. The results were shown as mean ± SD.

^b)^Averaged signal intensity of miRNA in the OHT-treated cells was quantified by microarray. The results were shown as mean ± SD.

**Table 2:** Retained miRNAs after tamoxifen treatment

miRNA	Controla)	OHTb)	OHT/Control
miR-134	1534.9 ± 350.9	16522.6 ± 8601.0	10.76
miR-549	8430.7 ± 4832.2	54010.0 ± 9153.1	6.41
Let-7a-3	3993.8 ± 1157.2	24562.5 ± 14098.6	6.15
miR-605	4200.2 ± 319.0	24924.5 ± 13551.3	5.93
miR-891b	123.6 ± 49.2	720.0 ± 370.9	5.82
miR-892	5339.9 ± 573.2	27856.3 ± 1230.8	5.22

^a)^Averaged signal intensity of miRNA in the vehicle-treated control cells was quantified by microarray. The results were shown as mean ± SD.

^b)^Averaged signal intensity of miRNA in the OHT-treated cells was quantified by microarray. The results were shown as mean ± SD.
