# Supplementary material for: MicroRNA-574-3p, identified by microRNA library-based functional screening, modulates tamoxifen response in breast cancer
Source: Sci Rep. 2015 Jan 6;5:7641. doi: 10.1038/srep07641 (PMC4284514; doi:10.1038/srep07641)
Supplement: Supplementary Information — Supplementary Figures [file srep07641-s1.pdf]

MicroRNA-574-3p, identified by microRNA library-based functional screening, modulates tamoxifen response in breast cancer

T Ujihira, K Ikeda, T Suzuki, R Yamaga, W Sato, K Horie-Inoue, T Shigekawa, A Osaki, T Saeki, K Okamoto, S Takeda, S Inoue

Supplementary Figures

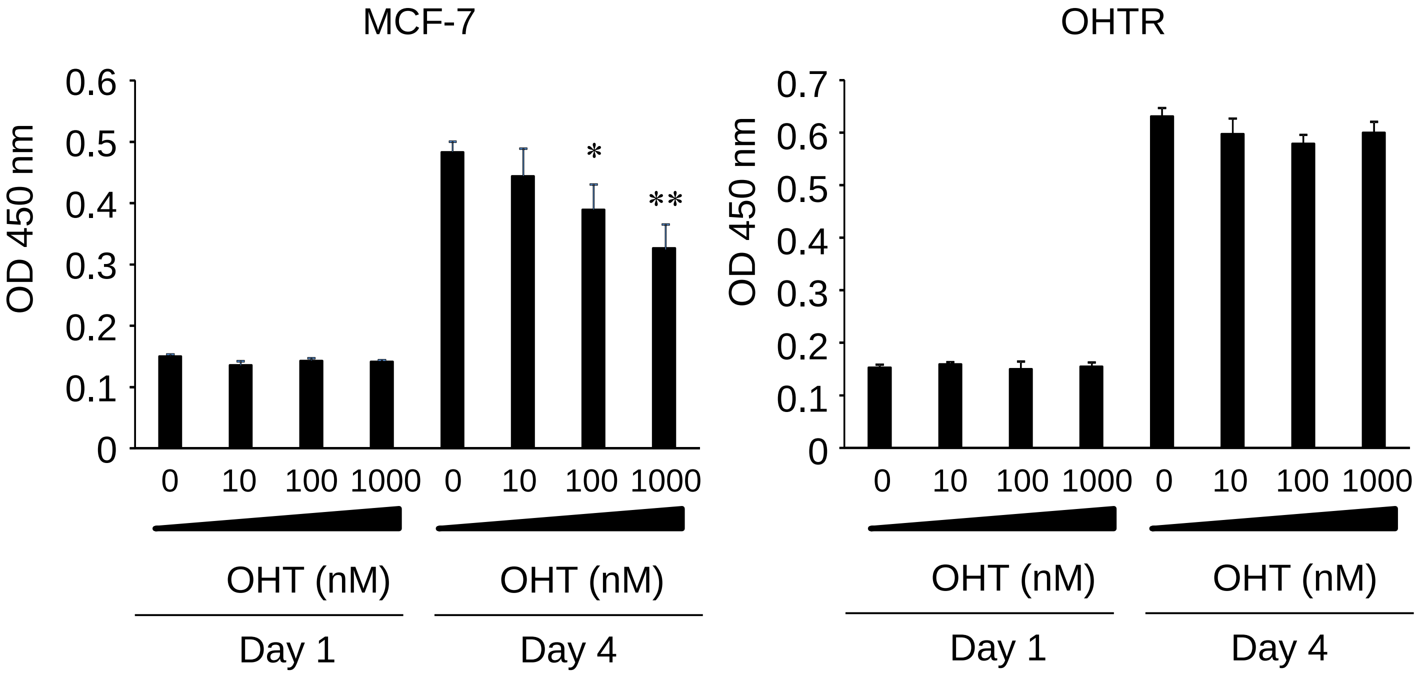

Supplementary Figure S1. Effects of tamoxifen on growth in MCF-7 and OHTR cells used. MCF-7 and OHTR cells were treated with 4-hydroxytamoxifen (OHT) at indicated concentrations. WST-8 cell proliferation assays were performed at the indicated time points. The absorbance values were measured using a microplate reader at 490 nm. Data are presented as mean ± s.d. (n = 3; *, $P < 0.05$; **, $P < 0.01$).
.

Supplementary Figure S1.
Ujihira et al.

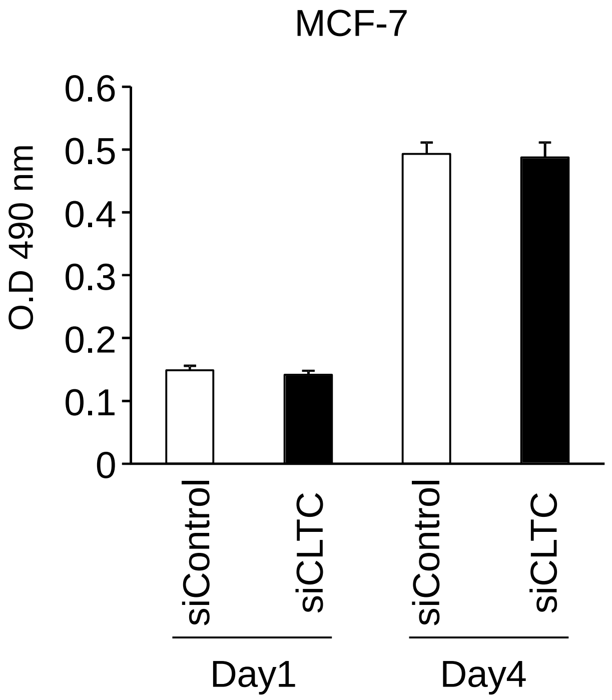

Supplementary Figure S2. Effects of siRNA targeting CLTC (siCLTC) on growth in MCF-7 in the basal condition. MCF-7 cells were transfected with siCLTC for 12 h (day 1) and then cultured in the medium without OHT. WST-8 cell proliferation assays were performed at the indicated time points. The absorbance values were measured using a microplate reader at 490 nm. Data are presented as mean ± s.d. (n = 3; *, $P < 0.05$; **, $P < 0.01$).

.

Supplementary Figure S2.
Ujihira et al.

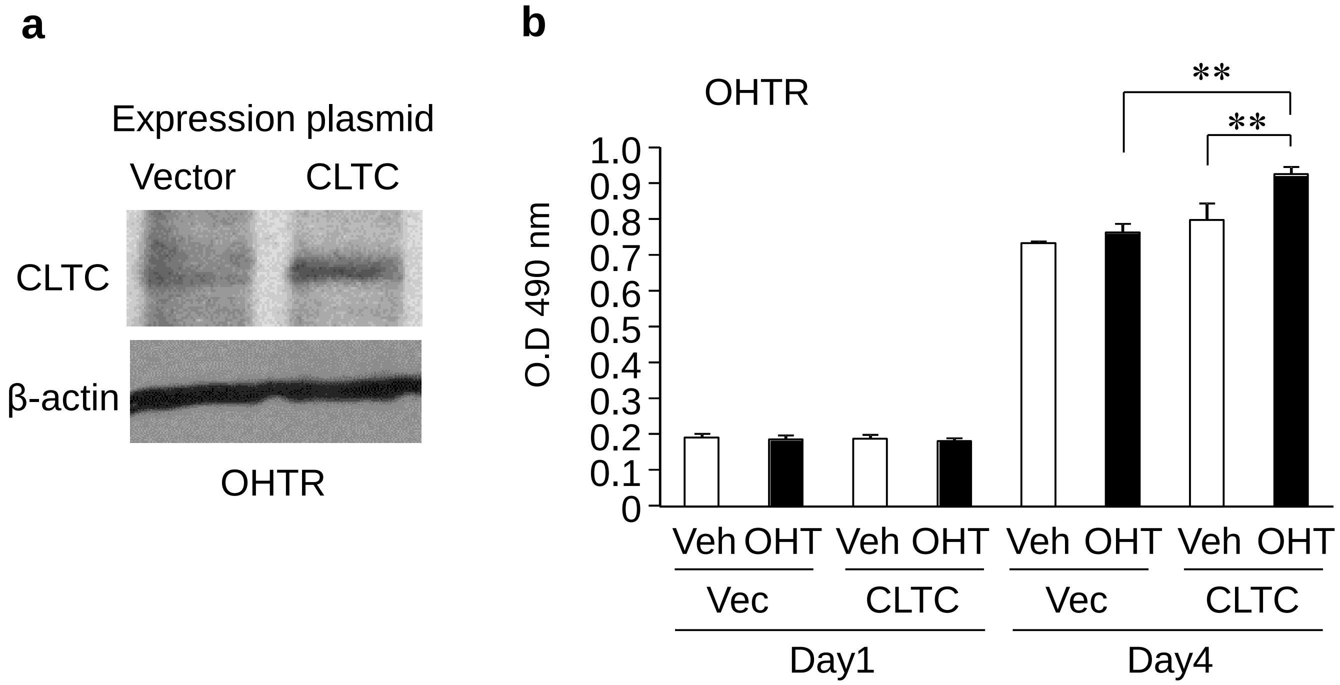

Supplementary Figure S3. Overexpression of CLTC stimulates growth of OHTR cells in the presence of tamoxifen. (**a**) Ectopic expression of CLTC in OHTR cells. OHTR cells were transfected with an expression plasmid for CLTC or empty vector for 48 h. Cell extracts were subjected to SDS-PAGE and western blot analysis using the CLTC and β-actin antibodies. (**b**) WST-8 cell growth assay of CLTC-transfected OHTR cells in the presence of tamoxifen. OHTR cells were transfected with an expression plasmid for CLTC or empty vector (vec) for 12 h (day 1) and then cultured in the medium containing 10^{-6} M OHT (OHT) or vehicle (veh). WST-8 cell proliferation assays were performed at the indicated time points. The absorbance values were measured using a microplate reader at 490 nm. Data are presented as mean ± s.d. (n = 3; **, *P* < 0.01).
.

Supplementary Figure S3.
Ujihira et al.
